# Impact of a cognitive stimulation program on the reading comprehension of children in primary education

**DOI:** 10.3389/fpsyg.2022.985790

**Published:** 2023-01-06

**Authors:** Claudia Reina-Reina, Pedro J. Conesa, Jon Andoni Duñabeitia

**Affiliations:** ^1^Centro de Investigación Nebrija en Cognición (CINC), Universidad Nebrija, Madrid, Spain; ^2^Facultad de Educación, University of Murcia, Murcia, Spain; ^3^Department of Language and Culture, UiT The Arctic University of Norway, Tromsø, Norway

**Keywords:** reading comprehension, academic performance, reading, cognitive training, executive functions

## Abstract

**Introduction:**

At present, numerous studies can be found in which influences and relationships between the principal executive functions, reading comprehension, and academic performance associated with reading are reported. However, there is still a lack of convergence regarding the impact of computerized cognitive training on children’s executive development and its transfer in academic reading performance and comprehension of written texts.

**Methods:**

This study analyzes the effect of implementing a cognitive stimulation program on the performance of reading comprehension and academic performance in the subject of Spanish Language and Literature. To this end, a total sample of 196 children from 23 educational centers received the cognitive intervention for 8 weeks, with three weekly sessions of between 15 and 20 min each occurring on non-consecutive days. Pre-test and post-test measurements were collected and analyzed.

**Results:**

The results demonstrate a significant increase in the reading comprehension scores. In addition, a significant impact of the training on the participants’ academic performance in the subject Spanish Language and Literature was found.

**Discussion:**

These results highlight the usefulness of computerized cognitive stimulation programs for reading comprehension enhancement.

## Introduction

Executive functions refer to several high-order cognitive processes mainly related with working memory, inhibitory control, and mental flexibility (Lezak, 1982; Miyake et al., 2000; Diamond, 2016). These processes are indispensable for controlling and regulating emotions, actions, and planned or intentional behaviors (Baggetta and Alexander, 2016). Likewise, they are essential to execute multiple tasks simultaneously and they are focused on achieving a specific objective autonomously and independently (Rosenberg, 2014).

Although the brain has developed up to 90% by the age of five (Blankenship et al., 2019), the maturation of the prefrontal cortex, an area that is specifically linked with these executive functions (see Hanakawa, 2011; Diamond, 2016), begins around the first year (Diamond, 1990), and does not end until the mid-twenties (Swaab, 2014). While this is true, it is not until approximately 3 years of age that we begin to observe a growth in cognitive development and frontal lobe activity (Moriguchi and Hiraki, 2013; Volckaert and Noel, 2015) that lasts until the last years of primary schooling. Hence, Primary or Elementary School represents a critical period for each child’s development of executive functions. This does not mean that this process stops once this period ends, but this development gradually decreases until adulthood (Conners, 2009; Roebers et al., 2014). For these reasons, the relevance of these cognitive skills in integral development and learning is indisputable, not only in children but also in adolescents and adults (see among many others Demagistri et al., 2014; Chen et al., 2016; Días and Seabra, 2016; Ober et al., 2019).

Traditionally, the scientific and educational community has focused on the study of the impact of executive functions on the reading skills of children and adolescents, given the importance of these skills as a central axis of many teaching-learning processes. The relevance of the reading processes in an educational system in which reading and writing play a crucial and central role is out of question, since a better reading efficiency and a higher level of reading comprehension are associated with better academic results. Furthermore, poor performance in reading processes has been linked to poor academic performance, ultimately being a potential cause of school failure. This scientific tradition continues nowadays, with numerous current studies demonstrating a close link between the comprehension of written texts and executive functions, specifically focusing on the decoding and recognition of words (Gough and Tunmer, 1986; Kamza, 2017; Nouwens et al., 2020; Ober et al., 2020). The cognitive operations necessary for the interpretation of the words’ meaning and function within the sentence, as well as the final understanding of the text, are mediated and influenced by the readers’ executive functions (Masson and Miller, 1983; Swanson, 1993, 1999; Swanson and Berninger, 1995; Piotrowska and Willis, 2019; Papadopoulos et al., 2020). Multiple studies have analyzed and evaluated the association between the principal cognitive skills and reading performance (Weerdt et al., 2013; Jacob and Parkinson, 2015; Peng et al., 2016; Sánchez-Pérez et al., 2018). These studies, together with others, emphasize the possible predictive effect of executive functions on subsequent school success (Diamond, 2013; Shaul and Schwartz, 2014), sometimes even more efficiently than IQ (Diamond, 2016; Johann and Karbach, 2019).

Hence, executive functions are inherently associated with complex learning processes such as reading [see the meta-analyses by Melby-Lervåg and Hulme (2013), Yeniad et al. (2013), Peng and Miller (2016) or Sala and Gobet (2017)]. There is a consensus that it is necessary, in order to carry out effective reading activities, to constantly change between the executive processes required for the recognition of the phonemes that make up each word, the understanding of the word’s meaning, its morphosyntactic and semantic function within a sentence, and its adaptation to the context in which it is being read. Simultaneously, throughout this process, it is necessary to inhibit and regulate the rest of the stimuli and/or activities irrelevant to the action being executed and preserve and remember the information read (Cartwright, 2012).

More specifically, findings regarding the link between working memory and reading processes suggest that children who have low working memory capacities show poorer performance in reading comprehension (Baggetta and Alexander, 2016; Butterfuss and Kendeou, 2018), or some of its subcomponents, such as phonological processes (Knoop-van Campen et al., 2018; Nouwens et al., 2020; Ober et al., 2020; Pérez-Pereira et al., 2020) or reading fluency (Kim and Wagner, 2015). Inhibitory control, another of the principal executive functions often linked to working memory (Miyake et al., 2000; Clair-Thompson and Gathercole, 2006; Diamond, 2013), has also been associated with phonological awareness and reading comprehension (Juhasz et al., 2003; Pylkkänen et al., 2004; McClelland et al., 2007; Conners, 2009). It is necessary to inhibit and discard the irrelevant information of the text that is being read to reach a global understanding of it (Weerdt et al., 2013; Iglesias-Sarmiento et al., 2015; Kamza, 2017; Lonigan et al., 2017).

Thus, considering the importance of executive functions for the acquisition and mechanization of reading processes, it is worth asking whether it would be possible to reinforce or promote these processes through a specific intervention on some components of executive functions. For this reason, and not surprisingly, the number of studies that report interventions based on cognitive stimulation programs to examine what effects they have on the performance of specific cognitive skills, and consequently, on subsequent academic performance and readership processes has increased exponentially in recent years (see Karbach et al., 2015; Söderqvist and Bergman-Nutley, 2015; Diamond, 2016). In this line, several authors have highlighted, suggested and evidenced how cognitive stimulation programs can have a positive impact on the performance of working memory and inhibitory control in children in Primary Education, and consequently, on their reading comprehension (Melby-Lervåg and Hulme, 2013; Peng and Fuchs, 2017; Siu et al., 2018; Nouwens et al., 2020; Conesa and Duñabeitia, 2021; Tapia and Duñabeitia, 2021). Nouwens et al. (2020) studied the contribution of executive functions to reading in a group of Dutch Primary School children (fifth graders) by using structural equation modeling to test the impact of scores in working memory, inhibition and planning tests carried out when the students were in fourth grade. Their results showed unambiguous contribution of the different executive functions to children’s reading skills 1 year afterwards, thus leading the authors to suggest that educational professionals aiming at developing intervention programs for reading comprehension skills “*should not only consider decoding and language skills children bring into the classroom but their executive functions as well*” (p. 186). In this line and in a very recent study, Conesa and Duñabeitia (2021) explored if a computerized game-based training program oriented at improving executive functions would affect academic performance in a large group of several hundreds of Spanish Primary School pupils. The intervention protocol took place over the course of 8 weeks, and when compared to a control group, the training group demonstrated improvements not only in different components of the executive functions, but also in children’s academic achievement in different school subjects.

Therefore, given the current panorama of research into the effects and associations that can be established between the principal executive functions and the reading processes, additional experimental data is needed in order to validate the idea that a cognitive intervention program based on executive functions can yield improvements in reading comprehension and related skills, as well as in the academic achievement dependent on reading processes. With this in mind, the present study pursues the main objective of analyzing the influence that a gamified program of cognitive stimulation can have on the performance of the reading comprehension and the academic success achieved in the subject Spanish Language and Literature in participants from 9 to 12 years of age. We tentatively hypothesized that an educational intervention based on implementing a cognitive stimulation program directed to cognitive functions in general, but with a strong focus on executive functions, would positively impact the achieved academic performance and the understanding of written texts.

## Materials and methods

### Design and participants

This study responds to a quantitative methodology, with a quasi-experimental longitudinal design. Participants corresponded to a single group and unique pre- and post-intervention evaluation measures were collected. The study stems from a tripartite collaboration between the Universidad Nebrija, Universidad de Murcia and CogniFit Inc. aimed at exploring the feasibility and efficacy of computerized cognitive training in different populations.

The study population was made up of a total of 196 participants. They were children of 4th, 5th and 6th grades of Primary Education (51.5% being girls), aged between 9 and 12 years old. The students belong to 23 different educational centers distributed across multiple provinces of Spain. As an exclusion criterion, we considered the presence of certain types of diagnosed learning difficulties or neurodevelopmental disorders or deficits, as well as physical disabilities that prevented participation in the proposed training or tests.

The selection and data collection of the respective participants was carried out following the criteria of the Research Ethics Commission of the University (Ref: 2989/2020) and Spanish Organic Law 3/2018 on the Protection of Personal Data. Additionally, participation in this study was entirely voluntary, as stated in the different information sheets shared with the families and educational professionals. Furthermore, the study was done with parental or legal guardian authorization using a signed consent form. All participants were informed that they could terminate their collaboration in the program at any time. Finally, participants were also informed of the confidential treatment of their data and that these were collected solely and exclusively for research purposes.

### Measurement instruments

A gamified cognitive stimulation program developed by CogniFit (CogniFit Inc., San Francisco, CA, USA) was used for this study. This tool allows the stimulation of five different domains (namely, reasoning, memory, attention, coordination, and perception) and has been shown to boost users’ executive functions by tailoring the training program in a personalized way, adapting the difficulty level for each participant based on an algorithm fed by an initial evaluation. This initial comprehensive cognitive evaluation consisted of the Cognitive Assessment Battery (CAB)™ PRO test,^1^ which provides a general cognitive score as well as specific scores in each of the five measured cognitive domains. The training protocol was tailored to each child’s specific cognitive profile given the outcome of the initial cognitive test and the continuous performance in each of the training sessions. This was made possible by CogniFit’s patented Individualized Training System™ (ITS) software that automatically detects and adjusts the difficulty for each person in every session thanks to the collection of a series of variables that are used to make decisions about the next activities and their level of difficulty. Thus, each individual child had a tailored protocol involving different cognitive tasks in the form of games that were selected from a pool of 41 possible games developed by CogniFit with the individually adjusted difficulty level, making the training experienced fully personalized.

More specifically, the program selected for implementation in this group of participants was composed of 41 cognitive different games designed to stimulate different cognitive skills. Among the gamified cognitive tasks selected for this intervention protocol, most of them directly related to different sub-components of executive functions, such as the games Minus Malus (working memory and shifting), Neuron Madness (inhibition and shifting), Lane Splitter (shifting and inhibition), Visual Crossword (working memory), Mouse Challenge (shifting), Digits (working memory), Match it! (inhibition), Reaction Field (inhibition and shifting) Bee Balloon (inhibition and shifting), Candy Line Up (working memory), Water Lilies (working memory), Penguin Explorer (inhibition), 3D Art Puzzle (working memory), Puzzles (working memory), Happy Hopper (inhibition), Sudoku (inhibition, shifting, and working memory), or Drive me crazy (shifting and inhibition), among others. A complete list of the 41 games can be found in the Appendix.^2^ It should be noted that the gamified cognitive stimulation program has been used and validated in multiple previous studies, such as those carried out by Horowitz-Kraus and Breznitz (2009), Peretz et al. (2011), Haimov and Shatil (2013), Preiss et al. (2013), Shah et al. (2017), or Conesa and Duñabeitia (2021). To familiarize teachers with the platform and its correct implementation in the classrooms, a brief initial training of 30 min was carried out by the first author and second authors of the current study.

On the other hand, for the measurement and evaluation of the dependent variables, a reading test was implemented using Cognition (De Leeuw, 2019). The selected reading test was the Progressive Linguistic Complexity Test (CLP [Complejidad Lingüística Progresiva]; Alliende et al., 2004). This standardized battery measures the level of comprehension of written texts in children between 6 and 14 years old, and it has been adapted, validated, and used in the Spanish scientific literature on different occasions (González-Trujillo Calet et al., 2014). It is composed of eight different levels, divided into two parallel forms each, which allow for test–retest designs without item repetition. The specific test levels used in this study varied in their composition between two and three texts, followed by a series of deductive inferential reading comprehension questions, with multiple choice answers and a single correct possibility. As described by the authors, these texts move away from children’s daily experiences, bringing them closer to scientific and literary topics appropriate to their age. The Cronbach alfa coefficient indicated by the authors of this test is 0.97.

Together with the results of the reading test, the results of the official school evaluations in the subject Spanish Language and Literature obtained at the end of the 1st and 2nd three-month period were collected. These evaluations were provided by the teachers using numerical values from 1 (insufficient) to 10 (outstanding), according to the provisions of Spanish Organic Law 8/2013, of December 9, for the improvement of educational quality. The evaluation follows the specific regulations from each Autonomous Community, being very similar to each other, establishing the objectives and minimum contents of the subject Spanish Language and Literature according to the cycle and academic year of Primary Education in which the participants are located.

### Intervention and procedure

Participating schools and individuals were recruited *via* social networks and email. CogniFit’s cognitive stimulation program’s platform was made available to the participating the educational centers once they agreed to participate. The intervention took place between January and February 2021. The intervention had a total duration of 8 weeks, with three to four sessions each week, preferably on non-consecutive days, and with an approximate duration of 15–20 min per session. Importantly, none of the participants who were part of the final sample completed less than 15 sessions, since this was an *a priori* set criterion for exclusion under the assumption that the outcome measures would not be sensitive enough to changes produced by a smaller number of training sessions. Note at this regard that participants completed the training sessions during the school hours in their classroom setting and with their reference group. Hence, there was a high homogeneity in the training time and number of sessions within each school group, and with the few exceptions of individuals that could not attend school repeatedly during the training period for medical reasons (*N* = 4), the minimum of 15 training sessions was accomplished by nearly the totality of the initial sample. The measurements before and after treatment (namely, the pre-test and the post-test) were carried out in early January and early March 2021, respectively.

## Results

We proceeded to the statistical analysis of the data collected in the study using the statistical software Jamovi. Two analyses were carried out using as dependent variables the percentiles obtained in the assessment battery for reading comprehension (CLP) and each student’s scores for academic performance in the subject of Spanish Language and Literature. A series of repeated measures ANOVAs were run with the two temporal moments (namely, the Test Moment factor, with the levels pre-test and post-test) as a within-child factor, and a series of co-variables added to explore the role of interindividual differences: Age (in years), Gender (male vs. female), Number of Training Sessions, Total Training Time (in minutes), and Socioeconomic Status. Socioeconomic status was measured using the MacArthur scale of subjective socioeconomic status (Adler et al., 2000). Families assess the socioeconomic context to which they belong with respect to the rest of their community using a 1-to-10 scale.

First, a descriptive statistical analysis of the dependent variables is presented. Table 1 presents the means and standard deviations of the sample of 196 participants in each of the dependent variables as well as in the relevant co-variables (namely, age, socioeconomic status, number of training sessions and total training time in minutes).

**Table 1:** Descriptive data for the dependent variables and co-variables of interest.

	Age	SES	Training Sessions	Training Time (mins)	Reading comprehension		Academic Achievement	
					Pre-test	Post-test	Pre-test	Post-test
Mean	9.96	6.36	23.1	192	55.3	62.9	7.49	8.04
Standard deviation	0.81	1.47	3.92	48.7	26.1	27.9	1.66	1.47

Age is reported in years. SES corresponds to the estimated socioeconomic status in a 1-to-10 scale. The number of training sessions and the number of minutes corresponding to the training time refer to the adherence to intervention. The reading comprehension scores refer to the mean percentiles in the reading test. Academic achievement corresponds to the score obtained in the subject Spanish Language and Literature.

**Figure 1:**
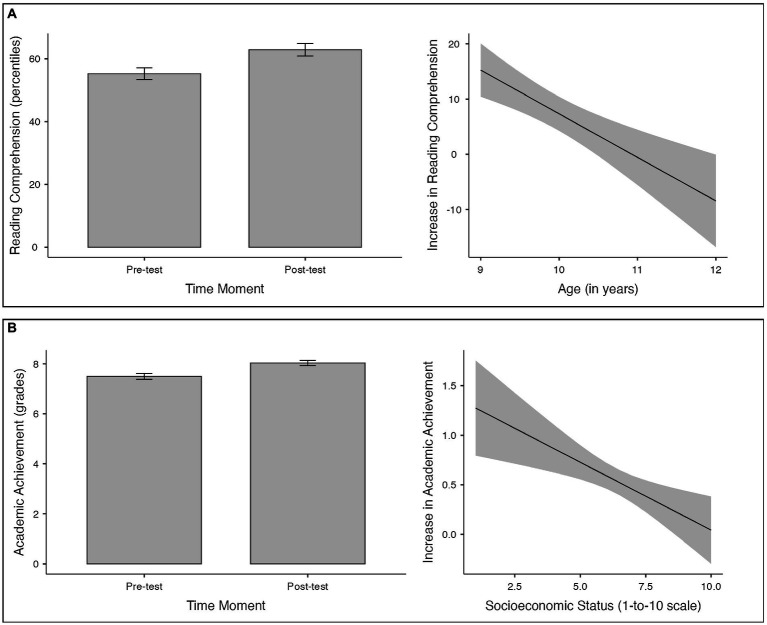
(Panel **A**) Mean reading comprehension level at pre-test and post-test in the CLP test (Panel **A**, left) and effect of the age of the participants as a moderator variable on the differential scores between pre-test and post-test (Panel **A**, right). (Panel **B**) Mean academic achievement at pre-test and post-test in the subject Spanish Language and Literature (Panel **B**, left) and effect of the socioeconomic status of the participants as a moderator variable on the differential scores between pre-test and post-test (Panel **B**, right). Means are presented with the 95% confidence intervals.

Second, these data were analyzed using an ANOVA test for repeated measures to check if significant differences existed because of the cognitive stimulation of executive functions. We found a significant increase in the percentiles obtained in the CLP test [*F* (1,190) = 14.61, *p* < 0.001, partial *η*^2^ = 0.071]. The only co-variable that modulated the effect was the age of the participants [*F* (1,190) = 16.05, *p* < 0.001, partial *η*^2^ = 0.078]: the effects of the training were most significant in younger boys and girls (see Figure 1A). None of the other co-variables were significant (all *F* < 1.25 and *p* > 0.26).

Likewise, for the dependent variable consisting of the grade indicating the academic achievement in the subject Spanish Language and Literature, the repeated measures ANOVA test showed a significant improvement in the scores after the cognitive stimulation intervention [*F* (1,190) = 5.72, *p* = 0.018, partial *η*^2^ = 0.029]. The only co-variable that showed modulating effects was the socioeconomic status [*F* (1,190) = 10.20, *p* = 0.002, partial *η*^2^ = 0.051]: the effects of training were greatest in boys and girls with lower socioeconomic status (see Figure 1B). None of the other co-variables were significant (all *F* < 1.54 and *p* > 0.21).

## Discussion and conclusions

The current study aimed to investigate the impact of a cognitive stimulation program based on a gamified training of executive functions on the performance shown by Primary Education Spanish children in the subject Spanish Language and Literature and on their reading comprehension. The starting point of the current research is that the different mechanisms related with reading processes pivot on different cognitive mechanisms that are domain-general, and that among them the executive functions play a fundamental role. This being the case and taking into account that executive functions can be effectively trained by means of computerized intervention protocols, this study hypothesized that an intervention on the inhibition and working memory components of executive functions would result in an improvement in reading competence.

Recent studies have highlighted the potential of computerized cognitive training for the development of children’s executive functions and many related components associated with academic achievement (Conesa and Duñabeitia, 2021) and decision-making (Sánchez-Castañeda et al., 2021). The results reported in this study demonstrate that children increase their reading comprehension performance after completing a cognitive stimulation program, suggesting that the implementation of gamified activities as part of a computerized cognitive training is a valid tool to improve children’s reading skills. Interestingly enough, these results align with previous research using the same platform for training certain executive functions in adults (Horowitz-Kraus and Breznitz, 2009; Shiran and Breznitz, 2010) and adolescents (Horowitz-Kraus and Breznitz, 2014), also demonstrating its impact in reading comprehension development.

An important aspect of the current results is the mediating role of the age of the participants, showing that the implementation of a gamified program for the cognitive stimulation of the executive functions related to working memory and inhibitory control results in a larger impact on the performance demonstrated in reading comprehension in younger than in older children. The evaluated participants in an age range between 9 and 10 years old showed a more significant increase in their reading competence after performing the intervention than the participants between 11 and 12 years old. Previous studies such as those carried out by Karbach et al. (2015) or Siu et al. (2018), in which the effect of a working memory training on the reading processes of children between 7 and 9 years old was analyzed, showed results similar to those found in the present study. In fact, in the present study the participants with ages between 11 and 12 years showed similar results in the two measurements carried out (pre-test and post-test), suggesting that the short and oriented cognitive stimulation program did not significantly improve their reading abilities as measured by the CLP test. This piece of evidence relates to the study by Peng et al. (2018), in which they defend how young readers, between 8 and 9 years old rely more on working memory, in contrast to more experienced readers who focus more on other memory-related processes to interpret and understand the text.

The present study also revealed how participants with a more disadvantaged socioeconomic background benefited to a greater extent from the computerized cognitive stimulation protocol in regard to their academic performance demonstrated in the subject of Spanish Language and Literature. This finding aligns with recent evidence reported by Weissheimer et al. (2019), who carried out a study with children between 8- and 10-years old belonging to different socioeconomic contexts, and showed that children of lower socioeconomic levels demonstrated a greater impact of a cognitive training program on their executive functions and reading performance. Likewise, Katz and Shah (2017) reported that participants between 6 and 18 years of age with low socioeconomic profiles showed more significant benefits derived from a cognitive intervention program, and a similar finding was reported by Gamino et al. (2014) with adolescent participants between 12 and 14 years of age from different socioeconomic strata. Altogether, these studies reinforce the idea of the great academic gap originated by socioeconomic differences between children and adolescents (Farah et al., 2006; Mackey et al., 2012), and they suggest that cognitive stimulation protocols could serve as a way of partially counteracting these social differences (Gamino et al., 2014; see also Sánchez-Pérez et al., 2018).

While these results demonstrate the educational value of computerized cognitive training programs oriented at enhancing executive functions, several cautionary notes should be made for future research. First, the use of a broader battery that evaluates children’s reading and reading-related cognitive skills and delves into their subcomponents would be desirable (see Locascio et al., 2010; Baggetta and Alexander, 2016; Georgiou et al., 2020). And second, the cognitive stimulation program was implemented in a single group that also constituted the unique sample of this study. In this line, it is worth noting that the present research is not the only one of its kind lacking a control group. Recent studies have highlighted the impact of computerized stimulation programs for the development of executive functions in reading skills in the whole study population without reporting control groups. Benefits of cognitive training on reading accuracy and fluency are observed in children aged 8–9 years (Horowitz-Kraus et al., 2019; Pasqualotto and Venuti, 2020). These findings are in line with similar results reported by Kerns et al. (2017), Passarotti et al. (2020), van der Donk et al. (2020), and Ramezani et al. (2021), where benefits in reading accuracy and fluency are observed in children aged 6–16 years following interventions dedicated to working memory development. Nonetheless, given the lack of a control group, it could be hypothesized that the difference in the outcome measures could have responded to reasons other than the cognitive training (e.g., an increase with developmental origin or due to extended experience with reading), and we acknowledge that the inclusion of a proper control group would have been desirable. Admittedly, a randomized controlled trial with two intervention arms (one of them being a control training) would represent the best scientific approach. We acknowledge this limitation of the current study. Future studies should be aimed at replicating these results with designs including control and experimental groups.

All in all, the present study provides the grounds for understanding the manner in which the malleability of the executive function system could represent a potential avenue to enhance reading comprehension. Preceding studies carried out with older children have suggested the need to explore this in younger samples of children who already do not have an effective mastery of reading processes (Karbach et al., 2015), and our data suggest that the impact of this type of intervention is most noticeable in novice readers who are still developing reading comprehension processes. Future studies should focus on younger samples as well as on those with atypical development, since computerized cognitive interventions have been shown to yield significant effects on participants who present an atypical development in reading or neuropsychological skills (Knoop-van Campen et al., 2018).

In conclusion, the present study showed the effect that the implementation of a cognitive stimulation program can entail in the reading comprehension performance and on the academic performance in children in the last years of Primary Education. Executive functions in children and their link with reading processes and the understanding of written texts have been widely studied in recent years, and building on this, the current study demonstrates the influence that essential co-variates such as ages or socioeconomic status can exert. The ability to understand written texts is a complex process that relies on a series of inherent cognitive and executive components. The importance of developing and training executive skills from an early age becomes evident, as well as the relevance of the early detection of difficulties or deficits in these cognitive functions. Computerized cognitive stimulation programs that adapt to the individual needs and characteristics of the children and adolescents can positively impact their academic success.

## Data availability statement

The raw data supporting the conclusions of this article will be made available by the authors, without undue reservation.

## Ethics statement

The whole protocol and data collection was carried out following the criteria of the Research Ethics Commission of the University (Ref: 2989/2020) and Spanish Organic Law 3/2018 on the Protection of Personal Data. Written informed consent to participate in this study was provided by the participants’ legal guardian/next of kin.

## Author contributions

All authors listed have made a substantial, direct, and intellectual contribution to the work and approved it for publication.

## Funding

This research has been partially funded by grants PID2021126884NB-I00 from the Spanish Government, ISERIE from Ayudas Fundación BBVA a Proyectos de Investigación Científica 2021, and H2019/HUM-5705 from the Comunidad de Madrid.

## Conflict of interest

The authors declare that the research was conducted in the absence of any commercial or financial relationships that could be construed as a potential conflict of interest.

## Publisher’s note

All claims expressed in this article are solely those of the authors and do not necessarily represent those of their affiliated organizations, or those of the publisher, the editors and the reviewers. Any product that may be evaluated in this article, or claim that may be made by its manufacturer, is not guaranteed or endorsed by the publisher.

## Footnotes

^1^ https://www.cognifit.com/cab

^2^While the focus of the training was on executive functions, it seems reasonable to assume that the series of games would also require additional resources from different cognitive domains, given the close relationship between videogames and general cognition. For this reason, a detailed analysis of the list of games presented in the Appendix suggests that the training extended to domain-general cognitive abilities: perception (visual scanning, estimation, auditory perception, spatial perception, visual perception, and recognition); coordination (hand-eye coordination, and response time); attention (divided attention, focused attention, inhibition, and updating); reasoning (shifting, planning, and processing speed); and memory (naming, short-term memory, contextual memory, working memory, phonological short-term memory, non-verbal memory, and visual short-term memory).

## Appendix

List of and links to the 41 games used in the training.

Bee Balloon: https://www.cognifit.com/brain-games/bee-balloon

Star architect: https://www.cognifit.com/brain-games/star-architect

Cube Foundry: https://www.cognifit.com/brain-games/blockout

Gem Breaker: https://www.cognifit.com/brain-games/gem-breaker

Gem Breaker 3D: https://www.cognifit.com/brain-games/gem-breaker-3d

Candy Factory: https://www.cognifit.com/brain-games/candy-factory

Candy Line Up: https://www.cognifit.com/brain-games/candy-line-up

Crossroads: https://www.cognifit.com/brain-games/crossroads

Color bee: https://www.cognifit.com/brain-games/color-bee

Digits: https://www.cognifit.com/brain-games/digits

Dragster Racing: https://www.cognifit.com/brain-games/dragster-racing

Fresh Squeeze: https://www.cognifit.com/brain-games/freshsqueeze

Fuel a Car: https://www.cognifit.com/brain-games/fuel-a-car

Jigsaw 9: https://www.cognifit.com/brain-games/jigsaw

Lane Changer: https://www.cognifit.com/brain-games/lane-splitter

Mahjong: https://www.cognifit.com/brain-games/mahjong

Mandala: https://www.cognifit.com/brain-games/mandala

Numbers line: https://www.cognifit.com/brain-games/number-lines

Minus Malus: https://www.cognifit.com/brain-games/minus-malus

Math Twins: https://www.cognifit.com/brain-games/math-twins

Match it!: https://www.cognifit.com/brain-games/match-it

Mouse Challenge: https://www.cognifit.com/brain-games/mouse-challenge

Visual crossword: https://www.cognifit.com/brain-games/name-me

Penguin Explorer: https://www.cognifit.com/brain-games/penguin-maze

Piece Making: https://www.cognifit.com/brain-games/piece-making

Puzzles: https://www.cognifit.com/brain-games/puzzles

3D Art Puzzle: https://www.cognifit.com/brain-games/3d-art-puzzle

Shore Dangers: https://www.cognifit.com/brain-games/shore-dangers

Happy Hopper: https://www.cognifit.com/brain-games/happy-hopper

Drive me crazy: https://www.cognifit.com/brain-games/simon-says

Slice and Drop: https://www.cognifit.com/brain-games/slice-and-drop

Neuron Madness: https://www.cognifit.com/brain-games/neuron-madness

Perfect Tension: https://www.cognifit.com/brain-games/perfect-tension

Sudoku: https://www.cognifit.com/brain-games/sudoku

Traffic Manager: https://www.cognifit.com/brain-games/traffic-manager

Twist it: https://www.cognifit.com/brain-games/twist-it

Water Lilies: https://www.cognifit.com/brain-games/water-lilies

Reaction Field: https://www.cognifit.com/brain-games/whack-a-mole

Butterfly Hunter: https://www.cognifit.com/brain-games/butterfly-hunter

Word Quest: https://www.cognifit.com/brain-games/word-quest

Words Birds: https://www.cognifit.com/brain-games/words-birds
